# The wide gape of snakes: A comparison of the developing mandibular symphysis in sauropsids

**DOI:** 10.1111/joa.70050

**Published:** 2025-10-02

**Authors:** Maricci Basa, Neal Anthwal, Ryan N. Felice, Abigail S. Tucker

**Affiliations:** ^1^ Centre for Craniofacial and Regenerative Biology, Faculty of Dentistry, Oral and Craniofacial Sciences King's College London London UK; ^2^ Department of Cell and Developmental Biology, Centre for Integrative Anatomy University College London London UK; ^3^ Department of Genetics Evolution and Environment University College London London UK

**Keywords:** collagen fibres, corn snake, dentary, feeding mechanics, Meckel's cartilage

## Abstract

The origin and evolution of snakes has been marked by the acquisition of many morphological and functional novelties, one of which is the possession of a highly kinetic skull allowing for the consumption of prey that are often larger than their head diameter. One feature of the iconic wide gape of macrostomate (large‐mouthed) snakes is due to changes in the rostral midline where the left and right hemi‐mandible come together. Across vertebrates, the two sides of the lower jaw are held together by the mandibular symphysis. In snakes, the two halves of the lower jaw do not fuse and the symphysis remains free, facilitating gape expansion. The symphysis has previously been explored in lizards and crocodiles, where ligamentous fibres and cartilages span the joint. Here, we compared the anatomy of the forming ‘free’ mandibular symphysis in the corn snake (*Pantherophis guttatus*) to symphysis development in two lizards, the veiled chameleon (*Chamaeleo calyptratus*) and the ocelot gecko (*Paroedura picta*), and an outgroup sauropsid, the chicken (*Gallus gallus domesticus*). Microcomputed tomography imaging, whole‐mount skeletal staining and histology staining confirmed the absence of bone and cartilage fusion at the mandibular symphysis in the corn snake during development, in contrast to the complete fusion of cartilage, but not bone, in both lizards and the fusion of the bone in the chick. Trichrome staining under circular polarised light and whole fast green staining highlighted that, while the symphyseal region was populated by a dense network of collagen fibres, the snake hemi‐mandibles were not connected across the rostral region by this fibrous network. Instead, collagen fibres extended backwards and around the snake mental groove to an intermandibular nodule. This nodule attached to the midline dorsally, allowing integration of the movement of the soft and hard tissues. Our analysis highlights the adaptations required to allow extreme lower jaw mobility and independence of the two sides of the jaw as found in macrostomate snakes.

## Graphical abstract

Our analysis explores the adaptations at the mandibular symphysis required to allow extreme lower jaw mobility in macrostomate snakes taking a comparative approach.

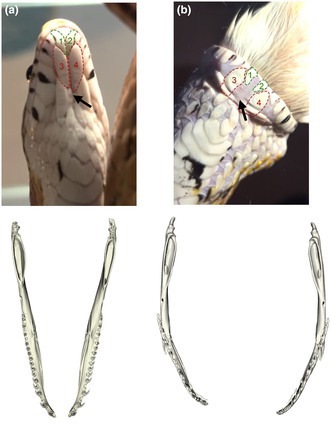

## 1 INTRODUCTION

The origin and evolution of snakes has been marked by the acquisition of many morphological and functional novelties, such as body elongation and loss of limbs (Cohn & Tickle, 1999; Gans, 1975; Head & Polly, 2015; Kvon et al., 2016; Sanger & Gibson‐Brown, 2004; Wiens & Slingluff, 2001) and high cranial and mandibular kinesis allowing for macrostomy (Da Silva et al., 2018; Kardong, 1977; Pandelis et al., 2023; Rhoda et al., 2021; Scanlon & Lee, 2000). Snake skulls have the highest degree of cranial kinesis among tetrapods (Gans, 1961). Snakes are divided into two infraorders: Scolecophidia and Alethinophidia. As snakes have adapted a sizeably elongated body, Scolecophidia and Alethinophidia have both evolved different ways of sustaining the resulting considerable mass. Through a comparatively small mouth, the plesiomorphic non‐macrostomate Scolecophidia consume vast numbers of prey at a rapid rate (Gans, 1961; Pough et al., 2015). In contrast, Alethinophidia consume fewer but dimensionally much larger prey (Pough et al., 2015). To allow for this, they have pronounced mandibular kinesis to support a wide lateral gape (Boltt & Ewer, 1964; Cundall & Greene, 2000; Gans, 1961; Greene, 1997; Kojima et al., 2020; Shine & Wall, 2008).

**FIGURE 1:**
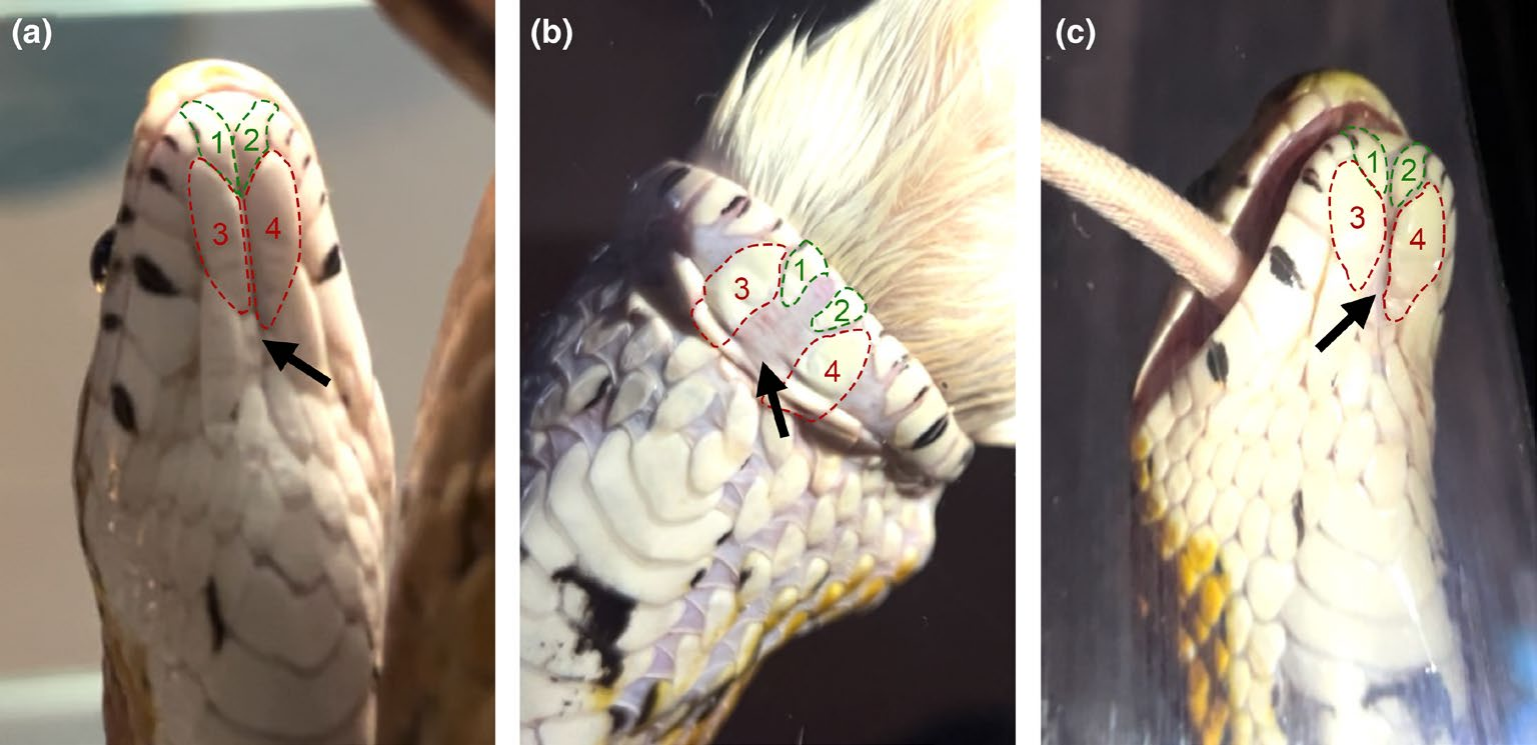
The mental groove allows for stretch during corn snake feeding. (a) Ventral head scale positioning at rest before feeding. (b) Ventral head scale positioning during active feeding. (c) Ventral head scale positioning immediately after feeding. Lower labial scales are labelled as ventral head scales 1 and 2 (green), while chin shield scales are labelled as ventral head scales 3 and 4 (red) (Hsu et al., 2017). The positioning of the mental groove is pointed out by the black arrows.

High mandibular kinesis in snakes is possible due to three novel and adapted joints that allow the lower jaw to move independently and aid unilateral feeding (Gans, 1961; Kley, 2001; Kley & Brainerd, 1999): (1) an extremely flexible quadrate‐articular joint (joining the lower jaw to the upper jaw); (2) an additional mobile joint between the tooth‐bearing dentary bone and the caudal compound bone, known as the intramandibular hinge; and (3) a highly extensible mandibular symphysis at the rostral midline. In addition to these skeletal changes, snakes possess an elasticated and multi‐pleated skin to support the mandibular structure and allow for large prey consumption (Close & Cundall, 2014). At rest, the skin lies within a longitudinal fold on the underside of the snake head, known as the mental groove (highlighted on the corn snake (*Pantherophis guttatus*) in Figure 1).

This work focuses on the mandibular symphysis. In most tetrapods, the two lateral halves of the mandible come together at the midline, although using a variety of mechanisms. The mandible can be united by fusion of the dentary bone, as observed in humans or by creation of a suture at the dentary or by fusion of the two rods of Meckel's cartilage which run throughout the lower jaw (Becker, 1986; Svandova et al., 2020). Crocodilians possess mandibular symphyses bridged by sutural ligaments and Meckel's cartilage (Holliday & Nesbitt, 2013; Lessner et al., 2019). Varanids have been reported to have both unfused dentary bones and unfused Meckel's cartilage at the symphyseal midline, with the two sides of the jaw connected by a fibrocartilage and fibrous tissues at the rostral tip (Holliday et al., 2010). The mandibular symphyses of non‐macrostomate Scolecophidia have been shown to retain a plesiomorphic morphology similar to lizards, presented as tight but unfused dentaries (Bellairs, 1984; Gans, 1961; Kley, 2001, 2006; Kley & Brainerd, 1999). The two arms of Meckel's cartilage were initially reported as fusing in Scolecophidia, but closer analysis showed that the two ends of Meckel's cartilage in fact did not fuse but were united in the midline by a cartilage and dense connective tissue (Cundall & Greene, 2000; Kley, 2006). This cartilage bridging Meckel's cartilage was evident in both Typhlopidae and *Leptotyphlops* blind snakes and was suggested to be composed of fibrocartilage rather than hyaline cartilage (Bellairs & Kamal, 1981; Kley, 2006).

The anatomy of the mandibular symphysis in macrostomate Alethinophidia is not as well documented, but a fibrocartilage at the midline has been suggested (Holliday et al., 2010; Kley, 2001). However, skeletal preparations of the developing Central African rock python (*Python sebae*) and hissing sand snake (*Psammophis sibilans*) highlight that the two arms of Meckel's cartilage do not meet at the midline, and there was no evidence of the development of an Alcian blue‐stained cartilage in the midline (Al Mohammadi et al., 2020; Boughner et al., 2007). Bellairs (1984) illustrated that the free mandibular symphysis in macrostomates was held together at the midline by lateral ligaments connecting the two rostral tips of the mandible to an intermandibular nodule (IMN) positioned at the midline further back in the jaw. This nodule is also referred to as the mid‐ventral raphe (Groombridge, 1979), the corpus musculo‐fibrosus (Kiran, 1981) or the fibrous interramal pad (Langebartel, 1968). Muscle fibres of the intermandibularis anterior (IMA) have been shown to run from the rostral tip of the dentary and backwards towards this nodule, controlling the stretch of the lower jaw (Cundall & Greene, 2000; Groombridge, 1979). In the common pipe snake (*Cylindrophis ruffus*), which has a relatively small gape size, two midline structures were described: a fibrocartilaginous interramal pad and intergular pad (Cundall, 1995). The intergular pad consisted of compact and bundled parallel‐running collagen fibres and was associated with the mental groove. The intergular pad of the common pipe snake had limited elastic properties and permitted only partial spreading of the dentary tips (Cundall, 1995). An elastic ligament directly connecting the left and right sides of the snake's lower jaw has been presumed in the literature (Greene, 1997; Hampton & Moon, 2013). For example, a ligament was noted running between the two arms of Meckel's in the Mexican burrowing python (*Loxocemus bicolor*) (Groombridge, 1979). However, whether this is also evident in snakes with pronounced intermandibular kinesis is unclear. As such, the composition of the mandibular symphysis in macrostomate snakes merits further investigation.

This work compares the microanatomy of the mandibular symphyseal gap in the developing snake, compared to that in other sauropsids of a similar age, via 3D reconstruction, imaging of collagen fibres and histological assays. Symphyseal structures were observed in late‐stage embryos and newborn hatchlings to follow the final development of this region prior to feeding and to identify any rudimentary structures that might be lost in adults. Two alternative hypotheses for the young snake mandibular symphysis were tested: the connection across the symphyseal gap between the two dentary bones is bridged by (A) a midline intermandibular nodule or (B) flexible ligaments running directly across the rostral midline.

In this study, we focus on the corn snake, *Pantherophis guttatus* (infraorder Alethinophidia, family Colubridae). The corn snake is a macrostomate member of the largest snake family, housing approximately 52% of all snake species (Figueroa et al., 2016). The corn snake is an oviparous non‐venomous constrictor, making it a potential lab model (Bealor & Saviola, 2007). In order to explore the snake mandibular joints within the context of other sauropsids, the symphysis was compared to that of the veiled chameleon (*Chamaeleo calyptratus*), the ocelot gecko (*Paroedura picta*), and an outgroup species, the domestic chicken (*Gallus gallus domesticus*). These species were selected due to availability of embryonic and hatchling tissue.

## 2 MATERIALS AND METHODS

### 2.1 Sample collection for experimental work

Sixteen embryos, hatchlings and juvenile snakes and lizards were sacrificed for symphyseal analysis (Table S1). A small breeding colony of *P. picta* and *P. guttatus* was housed at King's College London Biological Services Unit at Guy's Hospital and mated for breeding. *C. calyptratus* eggs were purchased from a breeder. Reptile eggs were incubated at 28°C on humid hatching substrate until hatching. Embryos and hatchlings were culled using schedule 1 methods as approved by the UK Home Office. All specimens were pithed to destroy the brain. Dissected heads were fixed in 4% paraformaldehyde and then processed through an ethanol series for storage in 100% ethanol or were fixed directly in 95% ethanol. Developmental staging of *P. picta* gecko embryos was based on Griffing et al. (2019). *G. gallus domesticus* chicken eggs were bought from Henry Stewart (Medeggs) and incubated at 37°C. Embryos were collected at embryonic days 10, 12 and 14 (developmental staging based on Hamburger & Hamilton, 1992), with E12 and E14 chicks ultimately used for analysis here. To image the snake jaw when fully extended, an 8‐week‐old snake was culled as above, but after pithing, the jaw was held open by wires at the intramandibular hinge during fixation (Figure S1).

### 2.2 Imaging the feeding snake

The change in gape during feeding was filmed in a corn snake (3‐year‐old male eating a defrosted dead mouse). Stills are shown in Figure 1. The snake was fed in a glass‐bottomed vivarium with a camera underneath.

### 2.3 3D reconstructions of sauropsid lower jaws

Lower jaws were dissected from their respective heads after fixing in 4% paraformaldehyde and washing in PBS. The lower jaws were scanned at King's College London, Centre for Craniofacial and Regenerative Biology using a SCANCO Medical microCT 50 scanner, using scan settings at energy/intensity 90 kV/88 μA, with voxel sizes from 4.4 μm to 6 μm. Additional chick samples were scanned at University College London, Centre for Integrative Anatomy using the Nikon XT H225 CT scanner, using X‐ray settings which ranged from 58 kV/138 μA to 68 kV/138 μA, with voxel sizes from 8.2 μm to 9.3 μm. Scan settings were optimised for each individual specimen. 3D segmentation of microCT scans was completed on Amira 3D 2021.1.

### 2.4 Histology

Lower jaws were dissected and decalcified in 0.5 M EDTA, before dehydrating in ethanol, clearing in xylene and embedding in paraffin wax. The samples were sectioned in frontal and transverse planes at 8 μm, then mounted onto glass slides. Sectioned tissues were stained using a standard Alcian blue/Sirius red staining technique for cartilage and bone, respectively, with haematoxylin for nuclear staining, using a Thermo Fisher Gemini AS automated slide stainer. Slides were viewed and imaged via standard light microscopy (histology) and polarised light microscopy (collagen birefringence) (Constantine & Mowry, 1968) using a Keyence digital microscope.

### 2.5 Skeletal preparation of corn snake heads

Corn snake heads were sacrificed and fixed in ethanol. Skin, brain and fat tissue were removed before placing in 95% ethanol to ensure proper dehydration. The samples were immersed in 100% acetone for further removal of adipose tissue for 2–5 hours, then for 3–5 days in fresh acetone depending on size. For staining cartilage, the samples were placed in an Alcian blue staining solution (0.3% Alcian blue 8GX in 70% ethanol), followed by bone staining in Alizarin red (0.1% Alizarin red S in 1% aqueous potassium hydroxide, KOH). After staining, the samples were cleared in 1% aqueous KOH. Once the samples were sufficiently cleared, they were placed in a glycerol:aqueous KOH series rising from 20% to 100% glycerol for storage at room temperature.

### 2.6 Fast green staining

Fine collagen fibre orientations at the mandibular symphysis were explored via fast green staining under anhydrous conditions (Timin & Milinkovitch, 2023). The durations of sample bleaching, KOH treatment and fast green staining were adjusted to accommodate for different jaw sizes, and nuclear staining was instead achieved using 1/1000 Hoescht +0.1% DMSO in PBST (1x PBS + 0.5% Triton X). The stained samples were placed onto a glass slide, and a well of petroleum jelly was created around the sample, ensuring no holes were present to avoid clearing agent dibenzyl ether (DBE) leakage. Two to three drops of fresh DBE were placed onto the enclosed sample; then, using a glass coverslip, the sample was covered and slightly flattened. Excess DBE was removed to assure a clean slide for confocal microscopy. The stained samples were imaged using a Zeiss LSM 980 confocal microscope. Image contrast was increased using image analysis via Fiji. Filters were applied to colour code the fibre by their depth/Z‐position.

## 3 RESULTS

### 3.1 Absence of hemi‐mandibular dentary or Meckel's cartilage fusion in the corn snake

**FIGURE 2:**
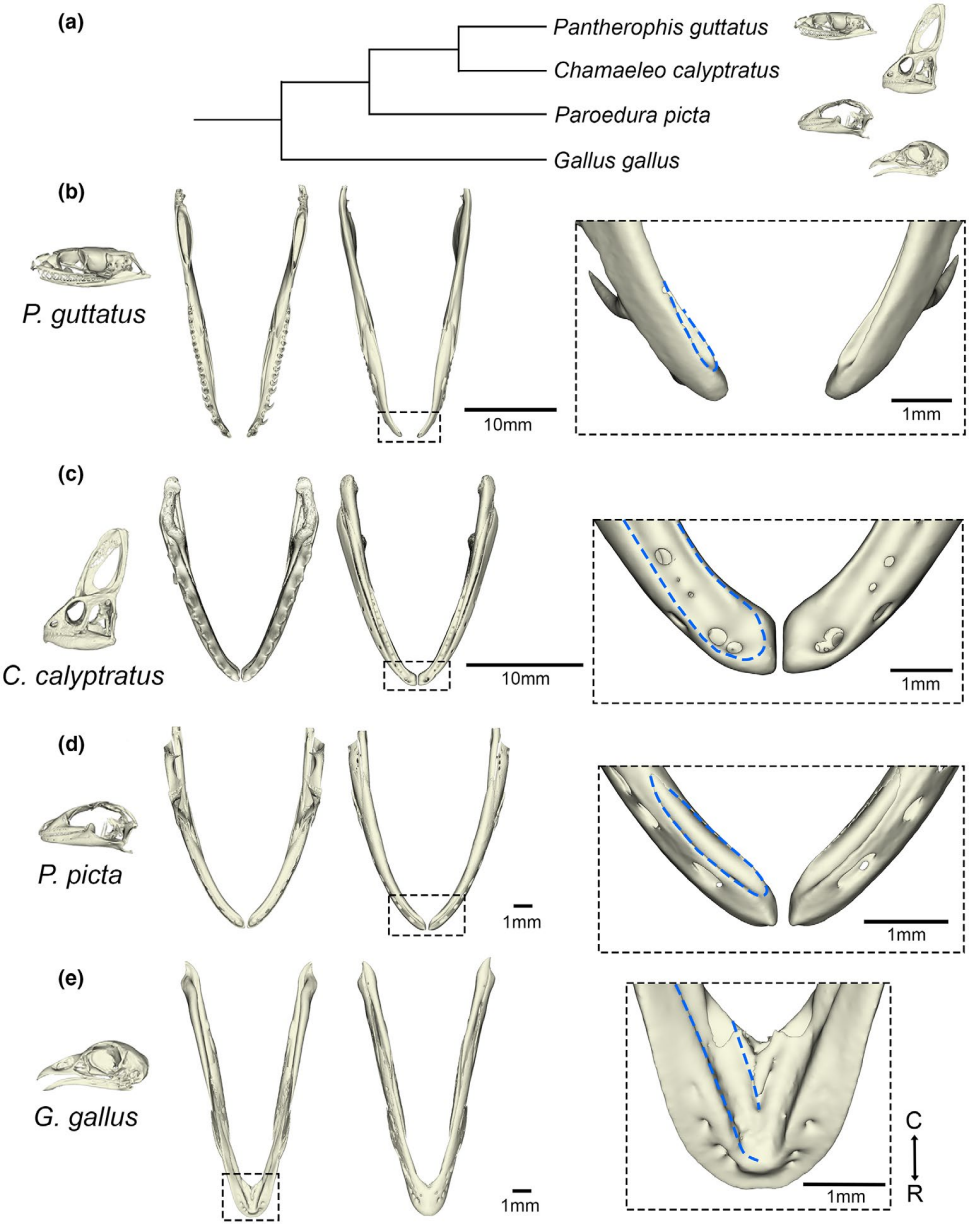
3D reconstructions of sauropsid lower jaws reveal dentary relationships. (a) A cladogram representing the relationship between the four sample species: *P. guttatus* (corn snake), *C. calyptratus* (veiled chameleon), *P. picta* (ocelot gecko) and *G. gallus* (chicken). (b) Dorsal (left) and ventral (right) views of the whole lower jaw of a newborn corn snake hatchling, highlighting the Meckelian groove (blue) on the lingual side of the dentary, which denotes the placement of Meckel's cartilage at the rostral tip. (c) Dorsal (left) and ventral (right) views of the whole lower jaw of a 2‐week‐old juvenile veiled chameleon, highlighting the rostroventral Meckelian groove on the dentary. (d) Dorsal (left) and ventral (right) views of the whole lower jaw of an E51 ocelot gecko, also highlighting the Meckelian groove on the rostroventrolingual side of the dentary. (e) Dorsal (left) and ventral (right) views of the whole lower jaw of an E14 domestic chicken, highlighting the presence of a symphyseal fusion at the rostral tip, thus displaying a singular fused lower jaw unit, and the Meckelian groove nested on the dorsal side of the rostral tip. Lower jaw orientation displayed from C = caudal end (upwards) to R = rostral tip (downwards).

Macrostomate snakes are known to have adopted a unique expansive gape. The expansion of the jaw during feeding can be appreciated by comparing the spacing of the mandible scales of the snake while at rest versus during active feeding (Figure 1a,b). The mental groove is evident at the midline of the snake's lower jaw and accommodates the excess skin between the midline scales (Figure 1b,c). The mandible returns to its original configuration immediately after feeding, suggesting active contraction (Figure 1c). A malleable mandibular symphysis allows for the wide lateral gape; however, the degree of skeletal liberation at this joint is unclear. To compare the anatomy of the dentary bone at the symphysis at rest, the two arms of the dentary bones were visualized in 3D by microCT in a selection of sauropsid species (Figure 2a). Agreeing with previous reports, the rostral ends of the corn snake dentary bones were unfused at the midline, creating a considerable osteological gap in the region of the symphysis (Figure 2b) (Al Mohammadi et al., 2020). The lower jaw of the veiled chameleon and ocelot gecko also showed unfused dentary bones at the midline, with a thin osteological gap separating the two sides of the jaw (Figure 2c,d respectively). In contrast, the dentary bones had fused at the symphysis in the developing chick's lower jaw (Figure 2e).

**FIGURE 3:**
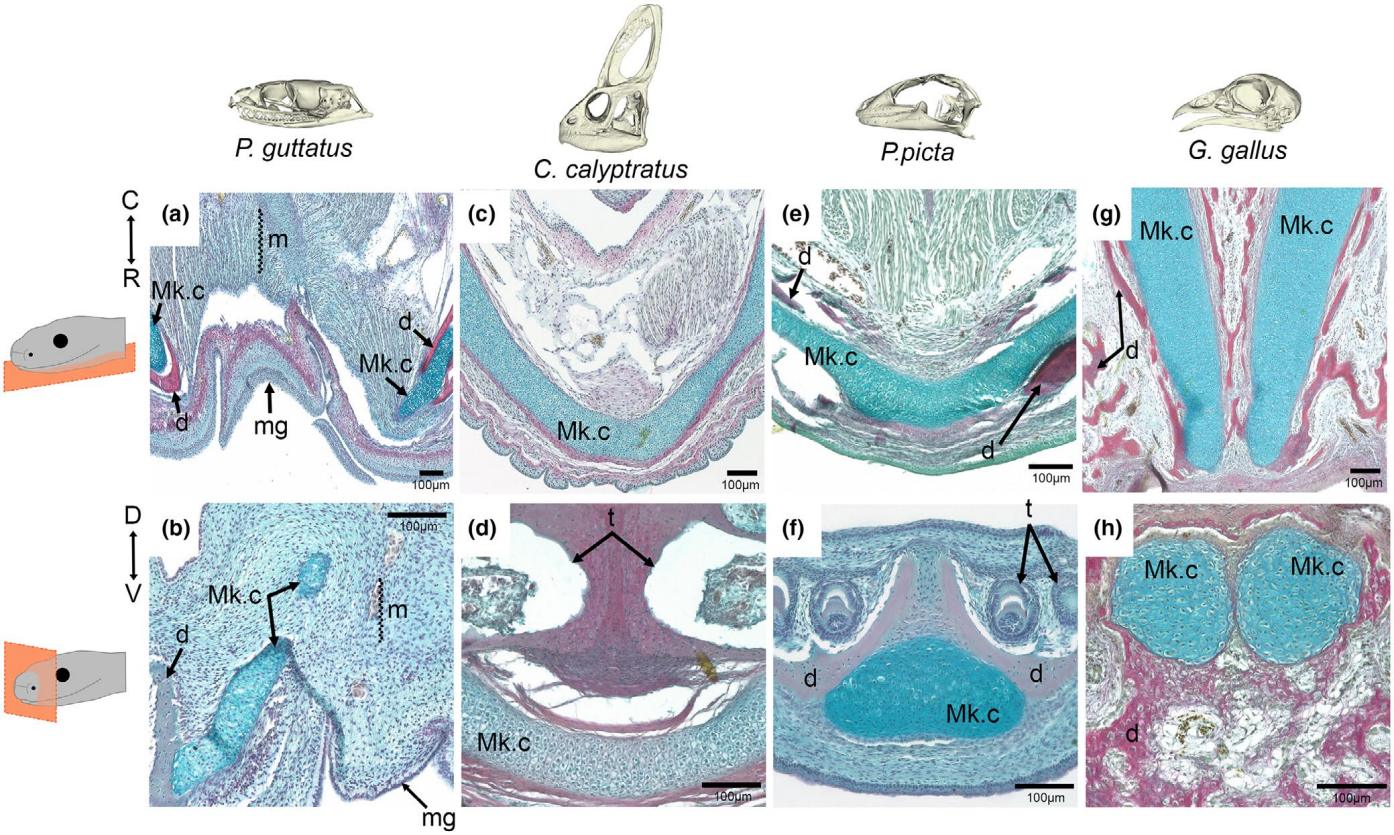
Cartilage arrangement at the rostral midline in embryonic and young sauropsids. Trichrome‐stained transverse (upper row) and frontal (lower row) sections of the mandibular rostra in developing sauropsids. (a) Transverse and (b) frontal sections from an E51 and pre‐hatching corn snake embryos (respectively), showing the presence of Meckel's cartilage (blue) at the rostral tip and its further rostral extension past the dentary (red) tip, although it does not join at the rostral midline, which location is signified by the position of the mental groove. (c) Transverse and (d) frontal sections from juvenile veiled chameleons, showing the fusion of Meckel's cartilage at the rostral tip. (e) Transverse and (f) frontal sections from an E51 and pre‐hatching ocelot geckos (respectively), showing the fusion of Meckel's cartilage at the rostral tip also. (g) Transverse E12 and (h) frontal E14 chick sections, showing the presence of Meckel's cartilage at the rostral tip of the lower jaw but failure to fuse at the rostral midline despite a fusion between the two sides of the dentary. Histological sections were stained with Sirius red (bone), Alcian blue (cartilage) and haematoxylin (nuclei and counterstaining). d, dentary; m, midline; mg, mental groove; Mk.c, Meckel's cartilage; t, tooth. Transverse section orientation displayed from C = caudal to R = rostral. Frontal section orientation displayed from D = dorsal to V = ventral.

**FIGURE 4:**
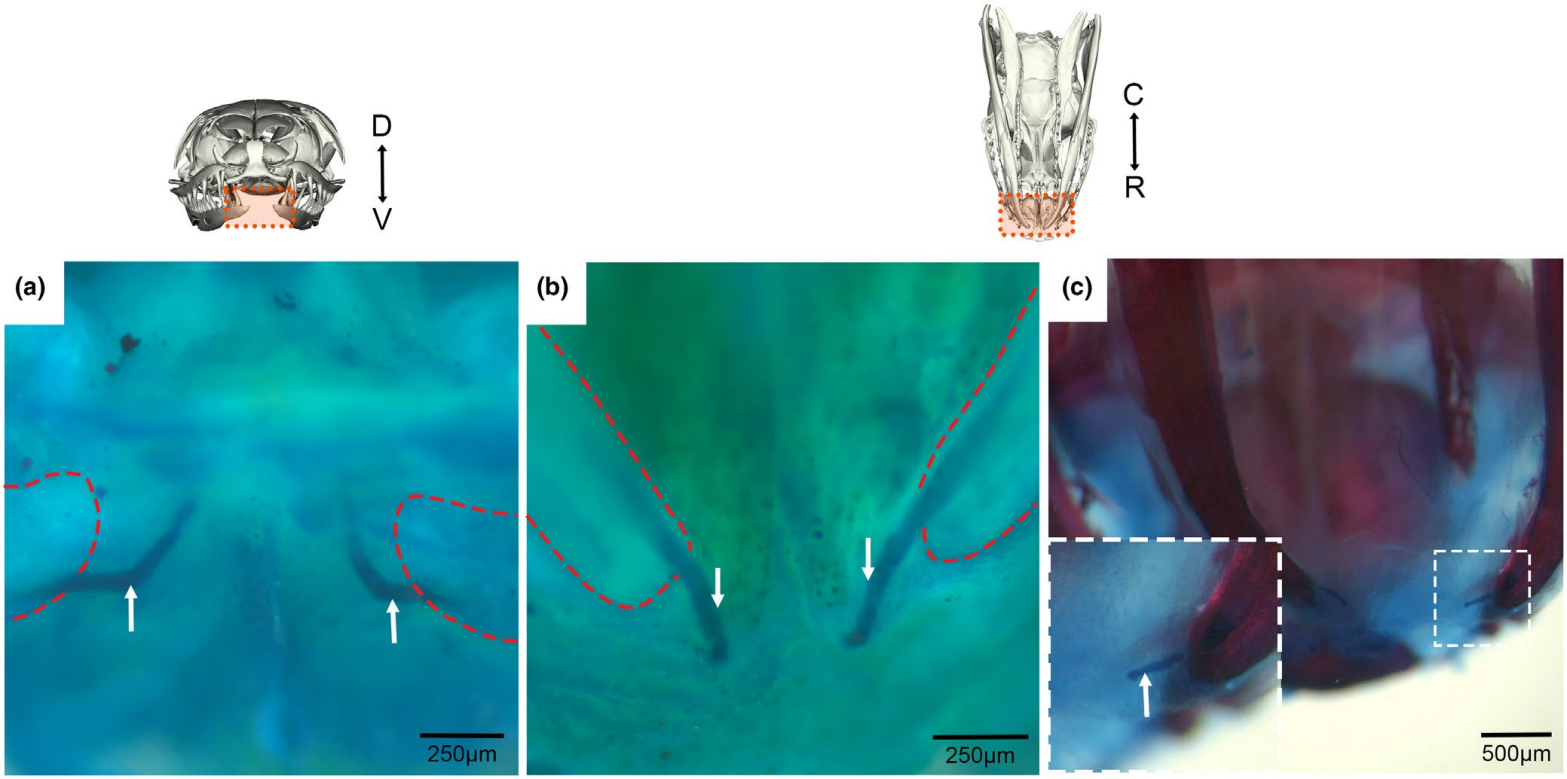
Cartilage arrangement at the rostral midline in embryonic and hatchling corn snake mandibles via whole skeletal staining. (a, b) A whole mount pre‐hatching corn snake embryo head stained with Alcian blue for cartilage detection. Meckel's cartilage (white arrows) is present at the rostral tip of the lower jaw which extends further rostrally past the dentary tip (outlined in red), although it does not join at the rostral midline, shown at frontal (a) and ventral (b) planes. (c) A whole mount newborn hatchling corn snake head stained with Alizarin red and Alcian blue for bone and cartilage detection (respectively) highlights the fineness of the rostrally extending Meckel's cartilage tip at the hatching stage. Frontal plane orientation is displayed from D = dorsal to V = ventral. Ventral plane orientation is displayed from C = caudal to R = rostral.

Cartilage is not visible by microCT without the use of counterstains, although the position of the cartilage within the dentary can be inferred from the presence of a groove in the dentary (Figure 2b–e). In order to visualize the cartilage arrangements at the symphysis, histology was performed in the four species. In the late‐stage embryonic corn snake, Meckel's cartilage extended rostrally past the dentary tip and curved upwards but did not join at the rostral midline (Figure 3a,b). In contrast, Meckel's cartilage was observed to bridge the gap in the 2‐week‐old veiled chameleon (Figure 3c,d) and late‐stage embryonic ocelot gecko (Figure 3e,f). Both lizard symphyses, therefore, have cartilage running across the midline uniting the two sides of the jaw. Interestingly, in the developing E12 (Figure 3g) and E14 chick (Figure 3h) lower jaw, the rostral tips of Meckel's cartilage extended towards each other at a very close proximity but did not join at the midline, despite the chick possessing a fused mandibular symphysis at this developmental stage. The relationship of the dentary and Meckel's cartilage in the corn snake was further assessed in 3D by skeletal prep (Figure 4). During late embryonic stages, Meckel's cartilage extended past the dentary (Figure 4a,b), curving upwards rather than towards the midline (Figure 4b). At hatching, this extension of Meckel's cartilage appeared as a slender projection, which, due to its size, was unlikely to have a major load‐bearing role (Figure 4c). No evidence of a cartilage bridging the two rods of Meckel's cartilage, as described in Scolecophidia, was observed in the corn snake by histology or skeletal prep.

### 3.2 Absence of direct fibrous hemi‐mandibular linkage in the corn snake

**FIGURE 5:**
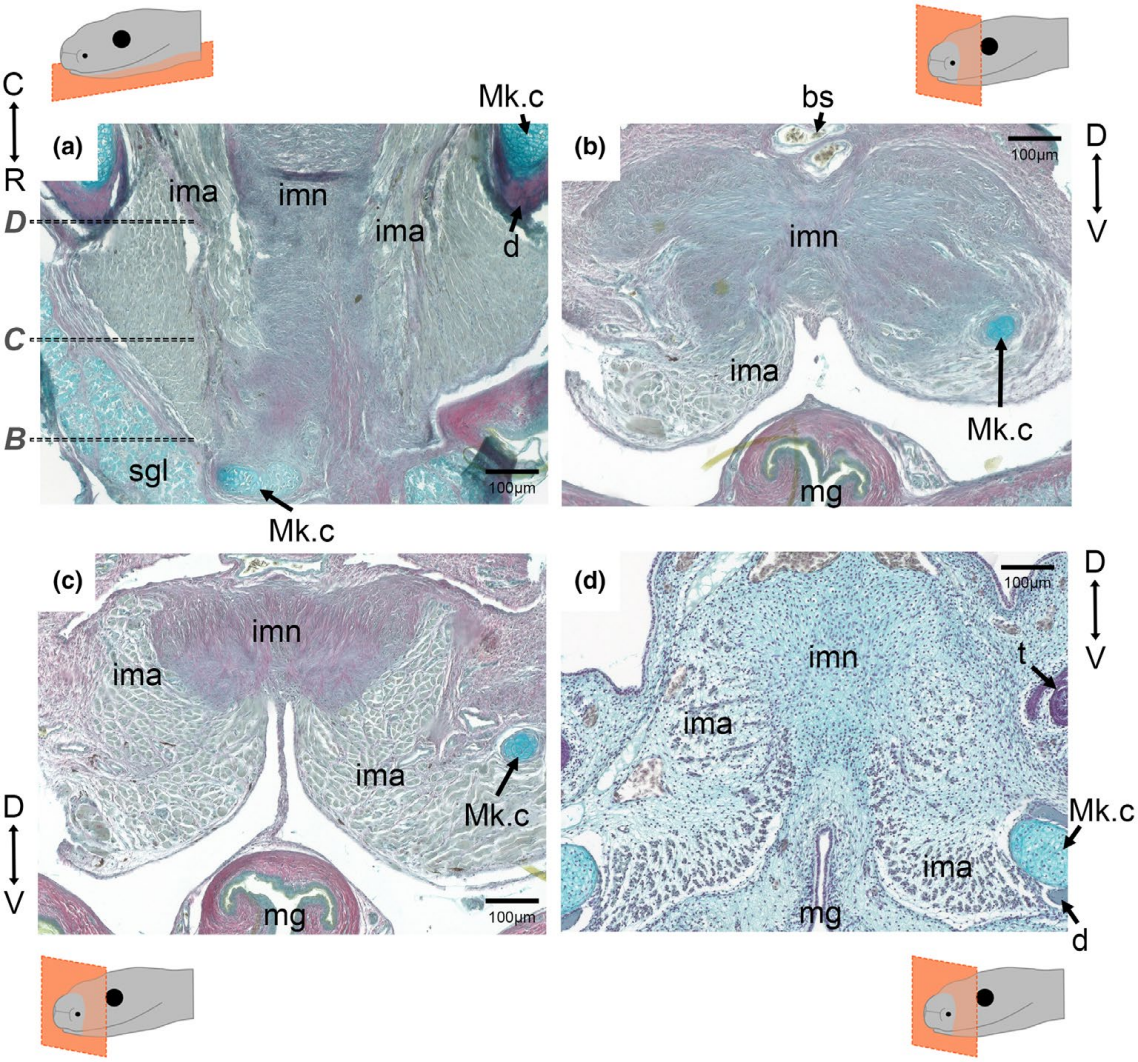
Relationships of the intermandibularis anterior muscle, intermandibular nodule and mental groove in the corn snake. (a) Transverse section of the rostrum of a near‐hatching corn snake, highlighting the plane of section shown in b, c and d. (b) Most rostral frontal section of near‐hatching corn snake. The intermandibular nodule is indistinct at the level. (c) More caudal section through the same specimen. The intermandibular nodule is more defined at this level with the intermandibularis anterior muscles connecting the hemi‐mandibular tips to the intermandibular nodule. A fine band of tissue connects the nodule to the mental groove. (d) Further caudal frontal section of the rostrum in a younger corn snake (E51), highlighting the connection of the hemi‐mandibles to the intermandibular nodule via the intermandibularis anterior muscles, but not directly to each other. bs, blood sinus; d, dentary; ima, intermandibularis anterior muscle; imn, intermandibular nodule; mg, mental groove; Mk.c, Meckel's cartilage; sgl, salivary glands; t, tooth. Transverse section orientation displayed from C = caudal to R = rostral. Frontal section orientation displayed from D = dorsal to V = ventral. Structure identification based on Bellairs (1984).

Macrostomate snakes have been proposed to possess an elastic connection at the mandibular symphysis, but the experimental evidence for this is lacking. To investigate this further, we investigated the developing symphysis in the corn snake at E51 by histology (Figure 5). In the most rostral region, Meckel's cartilage was closely associated with the intermandibularis anterior muscle (IMA) (Figure 5a,b) (Groombridge, 1979). More caudally, the IMA inserted on a region of condensed mesenchyme in the midline, which resembled the intermandibular nodule (IMN) as described by Bellairs (1984) (Figure 5c,d). The IMN was physically tethered to the tissue of the mental groove by a narrow band of connective tissue (Figure 5c). This would act to keep the mental groove aligned with the overlying nodule. Strikingly, a large space was evident under the muscle mass, which would have allowed the skin to move independently of the rest of the mandible at this rostral region (Figure 5b,c). Further back in the jaw, at the level of the tips of the dentary, the IMA muscle could be seen inserting into the midline nodule, running from the dentary (Figure 5d). At this position, the mental groove was now attached to the adjacent connective tissue. Partly due to the presence of the mental groove, there was no evidence of lateral ligaments running between the tips of Meckel's cartilage when viewed by histology.

**FIGURE 6:**
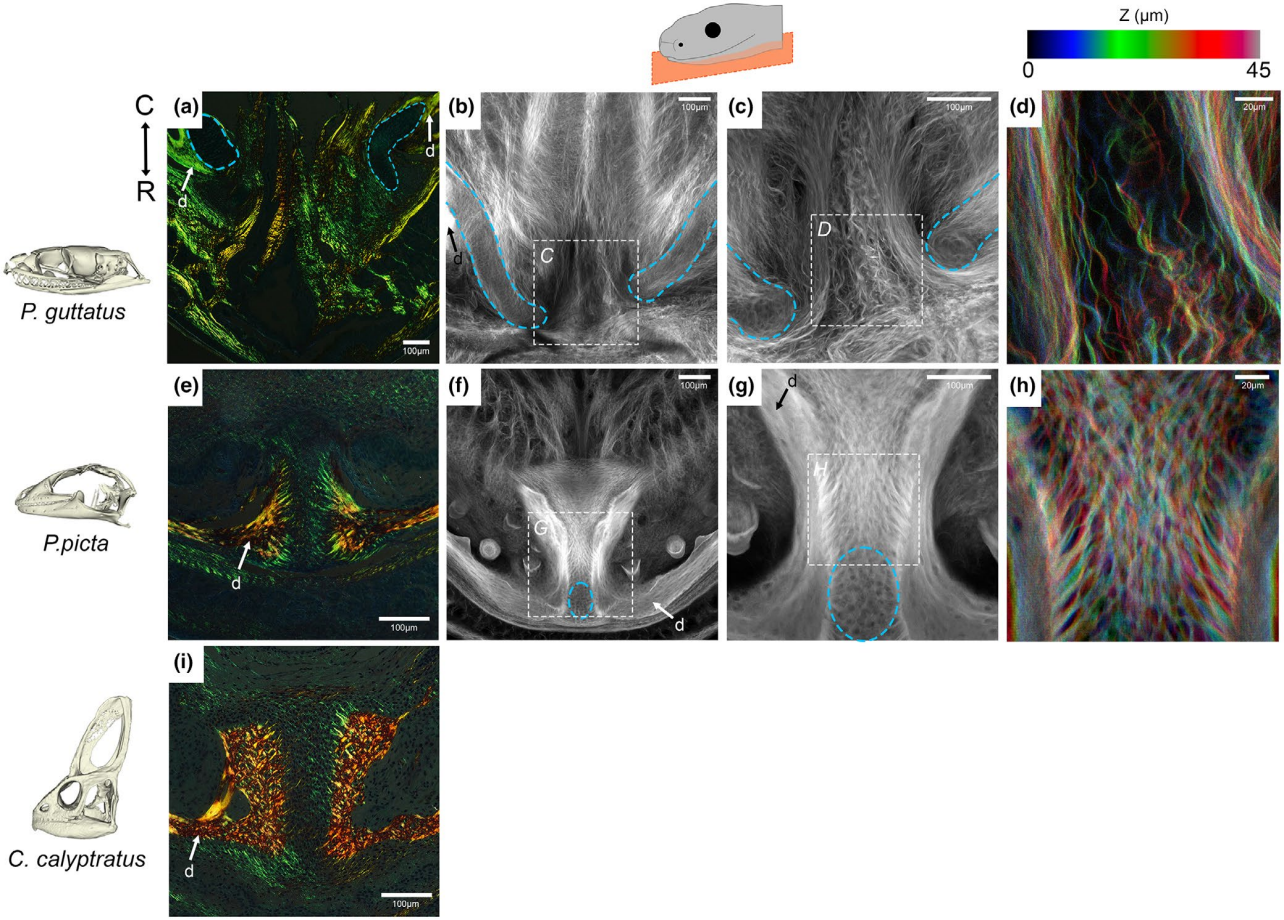
Collagen fibre orientation at the symphysis of embryonic sauropsids. The presence of collagen fibres was explored via the exploitation of collagen birefringence in trichrome‐stained transverse mandibular rostrum sections (a, e and i) viewed under circular polarised light. The fibrous profiles of the unfused snake (b–d) versus the fused gecko (f–h) symphysis were further explored via anhydrous fast green staining. (a) Mandibular symphyseal collagen in a newborn corn snake surrounded the dentary and cartilage (blue) tips but did not connect the two sides together. This was shown in clearer detail (b, c), where individual collagen fibres skirt the edges of the cartilage tips but do not provide contact between the two sides. (d) Filters applied to colour code the fibre by their depth/Z‐position. In contrast, collagen fibres in the (e) pre‐hatching gecko run across the symphyseal gap, thus connecting the two sides of the dentary. (f, g) This fibrous connection is shown in clearer detail, where collagen fibres bridge the two dentary tips at the mandibular symphysis of the gecko. (h) Filters applied to colour code the fibre by their depth/Z‐position. (i) The collagen fibres in the hatchling chameleon also run across and connect at the symphyseal gap. Transverse plane orientation displayed from C = caudal to R = rostral.

In order to investigate the arrangement of fibrous connections across the symphysis, the arrangement of collagen fibres was investigated using two techniques. Collagen birefringence was used to assess the patterns of collagen fibres in Sirius red‐stained sections in 2D (Figure 6a,e,i), while whole mount fast green staining was used to visualise the fibres in 3D (Figure 6b–d,f–h). In the corn snake, agreeing with the histology images, collagen fibres were observed running parallel to the length of Meckel's cartilage at the mandibular rostrum (Figure 6a). No collagen connection was observed between the hemi‐mandibles at the rostral tip of the corn snake. In contrast, the mandibular rostra of the embryonic gecko (Figure 6e) and young chameleon (Figure 6i) exhibited woven collagen fibres which ran across the mandibular symphyses, bridging their respective symphyseal gaps. In 3D, collagen fibres could be observed surrounding the tips of Meckel's cartilage at the snake symphyseal gap and did not bridge the two mandibular sides at the midline (Figure 6b,c). The fibres running parallel to the midline had a wavy morphology suggesting they were not under tension (Figure 6c,d). More posteriorly, some transverse fibres were evident inserting near the midline (Figure 6b). We used the same fast green imaging technique to view the developing symphysis in the embryonic gecko, which displayed fine collagen fibres connecting the dentary tips at the midline (Figure 6f,g). These fibres appeared under tension (Figure 6h), highlighting a clear difference in the jaw anatomy at the symphysis in the corn snake and gecko.

**FIGURE 7:**
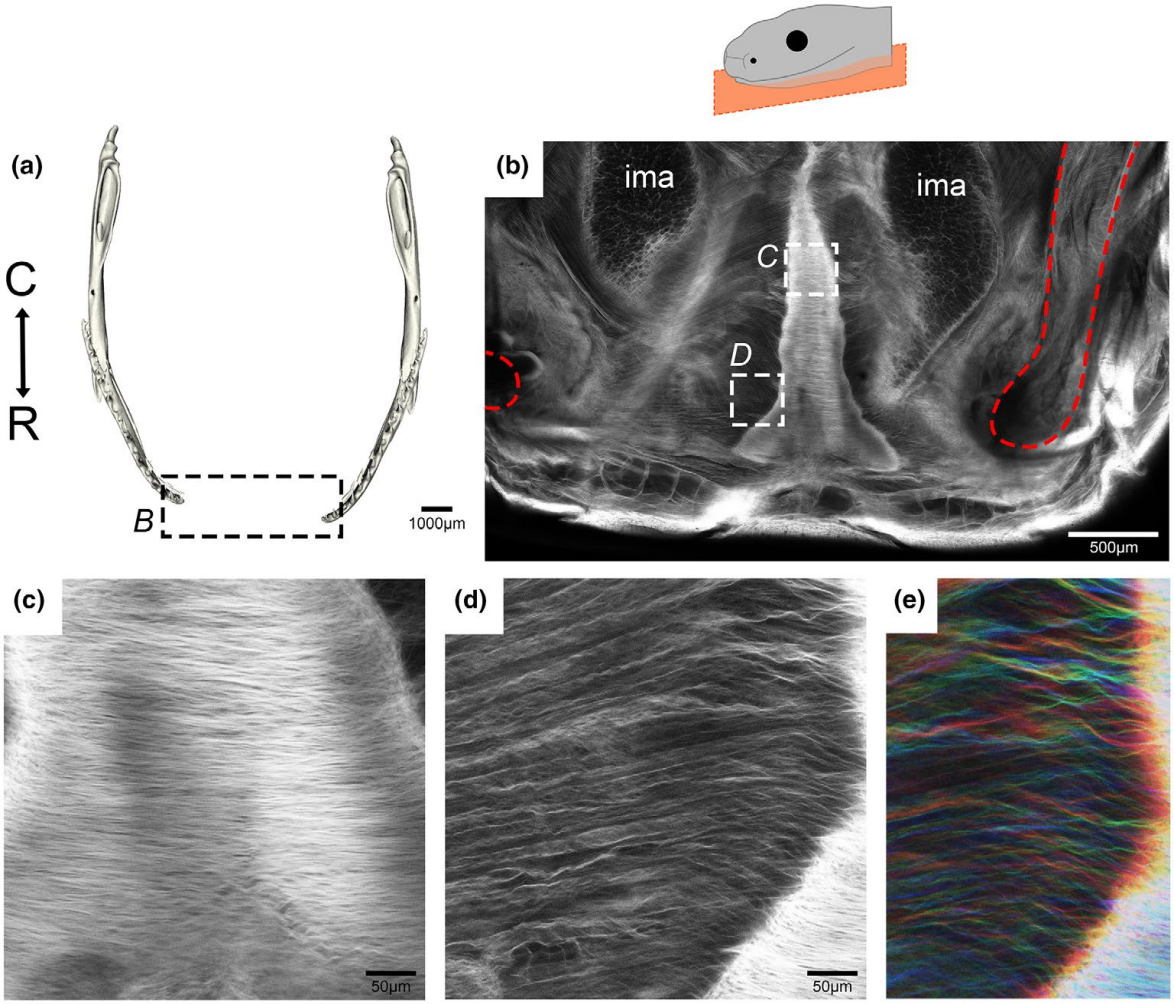
The anatomy and collagen fibre orientation at the symphysis of a ‘stretched’ corn snake mandible. The fibrous profiles of the stretched corn snake mandibular symphysis were explored via anhydrous fast green staining. (a) A 3D model dorsal view of the stretched lower jaw of an 8 week old juvenile corn snake. (b) A dorsal view of the fast green‐stained stretched jaw, highlighting the positions of the dentaries (in red), the intermandibular nodule and the intermandibular muscles. The dense collagen fibre network of the intermandibular nodule at the rostral midline (c) and the adjacent collagen fibres in between the intermandibular nodule and the left rostral dentary tip (d, e) are shown in greater detail. (e) Filters applied to colour code fibre by depth/Z‐position. ima, intermandibularis anterior muscle. Transverse plane orientation displayed from C = caudal to R = rostral.

The experimental work conducted above utilised snakes with a relaxed jaw positioning. In order to observe changes in fibre pattern during feeding, imaging was repeated on a corn snake that had had its jaws pinned wide immediately after culling (Figure 7a). Using whole mount fast green staining, the IMN was prominent in the midline, consisting of a dense network of parallel fibres (Figure 7b,c). From the IMN taut fibres were evident running towards the splayed dentary bones (Figure 7d,e), compared to the closed/resting jaw state of the same species (Figure 2b). The dentary bones are, therefore, supported by fibres that run from the IMN.

## 4 DISCUSSION

### 4.1 A comparison of the mandibular symphysis in sauropsids

**FIGURE 8:**
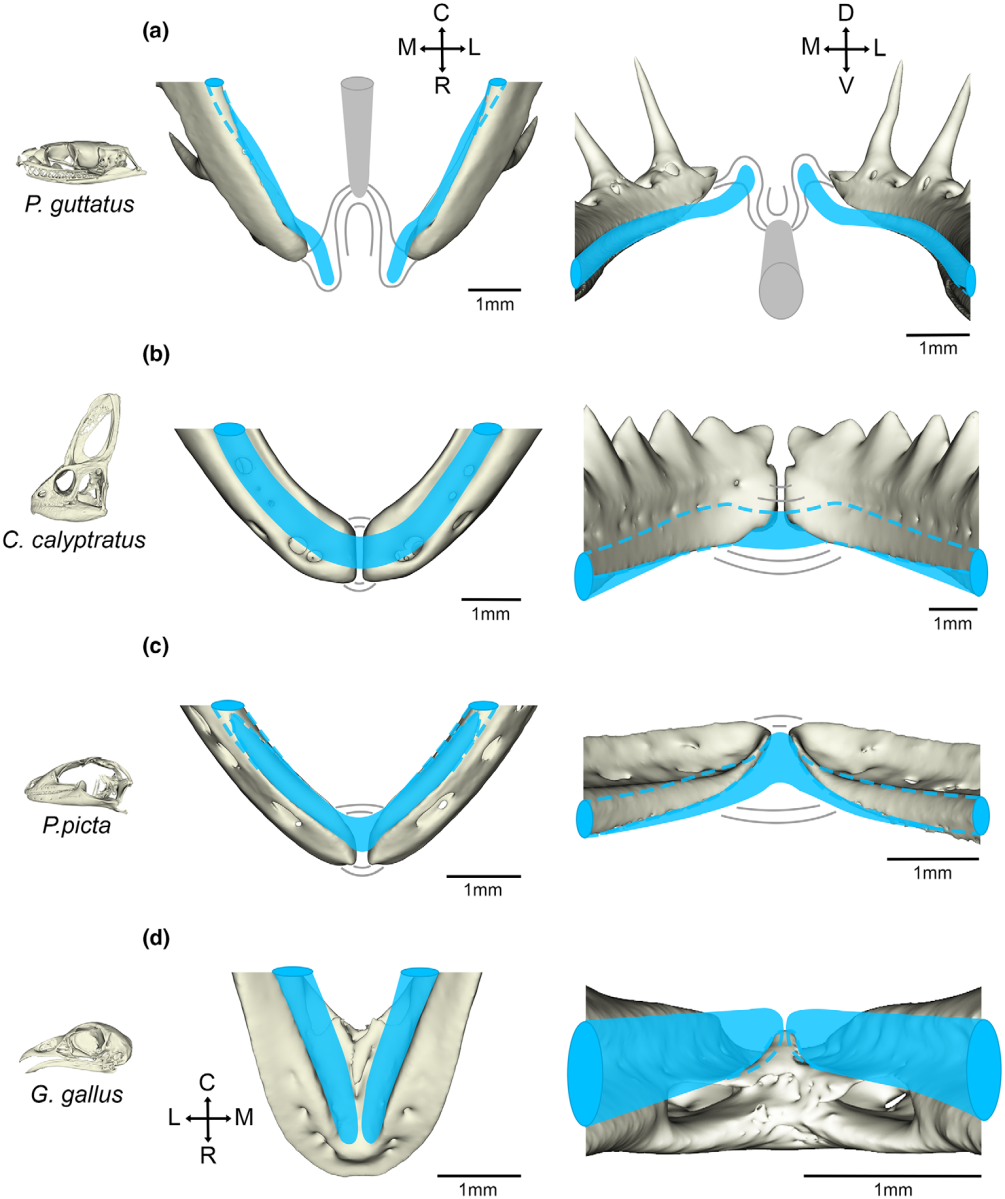
Schematic showing the relationships of the skeletal elements of the symphysis. The following structural elements at the rostral tip are displayed: The dentary bone (from microCT scan), Meckel's cartilage (blue), fibrous collagen network (grey) and intermandibular nodule depicted in A (solid grey structure). (a) Newborn corn snake hatchling mandibular symphysis at ventral (left) and frontal (right) views. (b) Two‐week veiled chameleon juvenile mandibular symphysis at ventral (left) and frontal (right) views. (c) Developing E51 ocelot gecko mandibular symphysis at ventral (left) and frontal (right) views. (d) Developing E14 chick mandibular symphysis at dorsal (left) and frontal (right) views. Transverse plane orientation displayed from C = caudal end to R = rostral tip and M = medial to L = lateral. Frontal plane orientation displayed from D = dorsal to V = ventral and M = medial to L = lateral.

Variations in the degree of liberation of the mandibular symphysis can be tracked through the sauropsid lineage (Figure 8). In the chick, the dentary bones fused at the symphysis during development, agreeing with the current avian literature (Bailleul et al., 2017; Choudhary et al., 2020; James, 2004; Prondvai & Stein, 2014). Fused dentaries are also found in birds with pouch‐like beaks, such as the pelican, which possesses flexible mandibular rami that allow large feeding volume capacity (Field et al., 2011; Meyers & Myers, 2005; Witton & Naish, 2013). Interestingly, Meckel's cartilage had not fused at the embryonic stages investigated in the chick, potentially as the surrounding fused dentary made further fusion of Meckel's cartilage redundant. In primates, a fused mandibular symphysis has been demonstrated to correlate to a stronger bite force (Lieberman & Crompton, 2000; Ravosa & Vinyard, 2020); however, a similar relationship is unlikely to be true in avians due to their pronounced streptognathy.

In contrast to the chick, the gecko and chameleon possessed an unfused dentary at the symphyseal midline, which would be predicted to allow some flexibility in this region. This has also been shown in the bearded dragon (*Pogona*) and monitor lizard (*Varanus*) (Holliday et al., 2010). Although the dentary bones did not fuse in the midline, a cartilaginous connection was evident in the two lizards analysed due to fusion of Meckel's cartilage across the midline (gecko, chameleon) (Figure 3c–f). This appears different from *Varanus*, where the rods of Meckel's cartilages do not fuse but are united by a distinct fibrocartilage at the rostral midline (Holliday et al., 2010; Torres‐Carvajal, 2003). A cartilaginous symphysis arrangement in lizards would be predicted to allow some degree of hemi‐mandibular mobility. Previous observations have shown that geckos move their prey in their mouths to aid in proper swallowing, and a kinetic jaw allows for better shock absorption when snapping their mouths (Bellairs & Carrington, 1966; Reilly & McBrayer, 2007). However, these same observations have noted that while some hemi‐mandibular mobility is present, fortified bites are evident and are as equally important for the consumption of harder prey (Bellairs & Carrington, 1966; Reilly & McBrayer, 2007). Fine collagen fibres were evident bridging the hemi‐mandibles at the rostral tips in the gecko and chameleon, agreeing with similar fibres shown in other lizards (Holliday et al., 2010).

In contrast, both the dentary bones and Meckel's cartilage were unfused in the corn snake. The degree of mobility was evident by comparing scans of the lower jaw at rest (Figure 2) and when stretched open (Figure 7). The presence of a fibrocartilage uniting the ends of Meckel's cartilage has been shown in Scolecophidia and has been proposed to be present in the common watersnake (*Nerodia sipedon*) (Holliday et al., 2010; Kley, 2006). No evidence was observed of a cartilage forming between the two rods of Meckel's cartilage in the corn snake from histology or skeletal preps, agreeing with previously published skeletal preps (Al Mohammadi et al., 2020; Boughner et al., 2007). It is possible, however, that an early condensation formed in this region, but never chondrified. This could be tested by assessing the expression of the chondrocyte master regulator *Sox9* at earlier stages. A connecting fibrocartilage may, therefore, have been lost during the evolution of macrostomate snakes, as a step towards a large gape. The potential nodule observed in *N. sipedon* (Holliday et al., 2010) may have been the more caudally positioned intermandibular nodule.

### 4.2 The microanatomy of a compliant mandibular symphysis in a macrostomate snake

In addition to the absence of a hard tissue connection at the midline, there was no evidence of any ligamentous tissue running directly tip to tip between the two ends of Meckel's cartilage, as often portrayed in snake feeding literature. In fact, this would seem unlikely to occur in any snakes with a pronounced mental groove, where space is required for the infolding of the midline skin. Some transverse fibres might be located more ventrally in the jaw but not attach to the dentary and Meckel's cartilage themselves. Instead, the muscles and collagen fibres were shown to run parallel to the midline, not across it in the corn snake at rest (Figure 5). The collagen fibres were wavy in the resting position and taut in the stretched jaws, mimicking the normal changes during feeding. The hyperextensible IMA muscles (Close et al., 2014) attached to the dentary and continued backwards to insert on the intermandibular nodule (IMN). This nodule was physically tethered to the mental groove by a narrow region of connective tissue, spanning a noncellular region (Figure 6). This arrangement would allow coordinated movement of the skin of the mental groove and the midline connective tissue, while allowing some independent expansion.

The IMN of the reduced gape pipe snake has been described as fibrocartilaginous, sitting on a pad of compact collagen fibres (Cundall, 1995). In vipers, in contrast, the nodule was described as composed of dense fibrous tissue (Bellairs, 1984). Similarly, the IMN in the corn snake appeared to be composed of a dense meshwork of fibres (Figure 7). The nodule did not strongly express Alcian blue at any of the stages analysed, and the cells did not have the rounded appearance characteristic of cartilaginous cells under histology. It would therefore be useful to provide some molecular analysis in the corn snake to identify the cell type of the IMN. The fibrous IMN in the hatched corn snake appeared similar to the intergular pad described in the pipe snake (Cundall, 1995). During the evolution of macrostomy in alethinophidian snakes, the composition of this nodule might have shifted from fibrocartilaginous to fibrous in order to provide greater elasticity at the midline region.

The anatomical arrangement of muscles, collagen fibres, midline nodule and mental groove would be predicted to prevent overstretching and aid in rapid retraction of the two sides of the jaw, as observed after feeding (Figure 1). A number of changes to the soft tissues at the symphysis would, therefore, have been necessary to accommodate the loss of connection of hard tissue at the midline. Future work could involve functional testing of the different components of the symphysis. For example, the role of fibre pattern could be tested by disrupting collagen fibre cross‐linkage to assess the impact on jaw mobility. Pharmacological inhibition in reptile embryos has recently been successfully carried out in ovo, while snake tissue explants have been cultured to manipulate ex vivo (Gaete & Tucker, 2013; Santos‐Durán et al., 2025), providing potential methods for carrying out manipulations.

## AUTHOR CONTRIBUTIONS

Credits to Abigail S. Tucker and Ryan N. Felice for the concept and design of the project; Maricci Basa for the acquisition of the data; Maricci Basa and Abigail S. Tucker for drafting the manuscript; and Maricci Basa, Abigail S. Tucker and Neal Anthwal for the interpretation of the data. All authors are credited for the critical revision and approval of the manuscript.

## CONFLICT OF INTEREST STATEMENT

Abigail S. Tucker is president elect of the Anatomical Society. The other authors declare no conflict of interest.

## Supporting information


Table S1.

## Data Availability

The data that support the findings of this study are available from the corresponding author upon reasonable request. microCT Scans used in this paper are available from Morphosource (Morphosource.org).
