# Supplementary material for: The wide gape of snakes: A comparison of the developing mandibular symphysis in sauropsids
Source: J Anat. 2025 Oct 2;248(6):937–49. doi: 10.1111/joa.70050 (PMC13148632; doi:10.1111/joa.70050)
Supplement: Supplementary file 1 — Table S1. [file JOA-248-937-s001.docx]

Supplementary table 1. Ages of sauropsid samples for experimental analysis.

| Sampled sauropsid | Analysis | | | |
| --- | --- | --- | --- | --- |
|  | microCT imaging | Histology under normal and polarised light  (transverse; frontal) | Whole mount skeletal preparation | Fast green staining |
| Corn snake | Newborn hatchling and 8 week old juvenile | E51;  pre-hatching embryo; pre-hatching embryos (x3 for Fig. 5) | Pre-hatching embryo, newborn hatchling | Newborn hatchling x2  8 week old (x1 for relaxed/closed lower jaw, x1 for stretched lower jaw) |
| Veiled chameleon | 2-week juvenile | Newborn hatchling;  2-week juvenile | / | / |
| Ocelot gecko | E56 | E51; pre-hatching embryo | / | Pre-hatching embryo |
| Chicken | E14 | E12; E14 | / | / |

Each listed specimen was used in quantities of x1, unless stated otherwise.


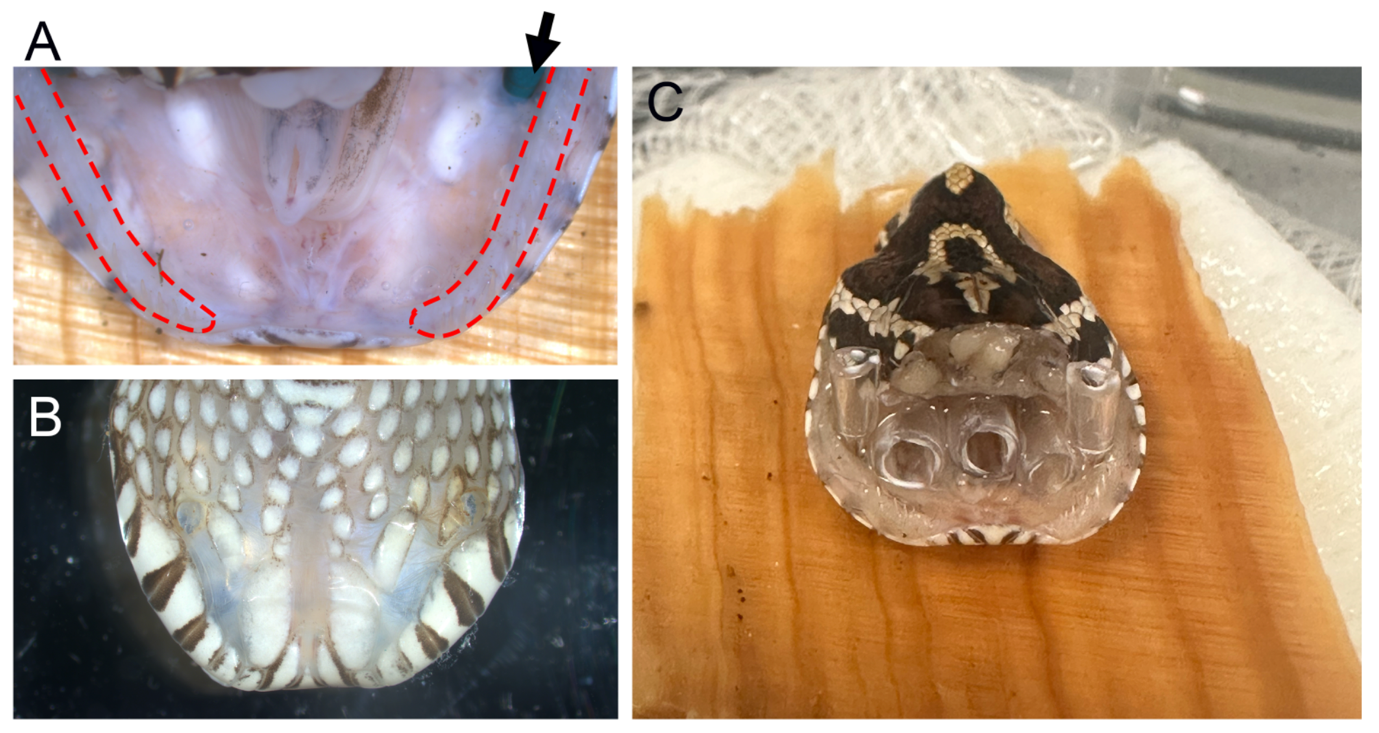


Supplementary figure 1. Corn snake stretched jaw surface anatomy and preparation.

(A) Initial pinning of the snake lower jaw using metal paper clips (arrow) and a small wooden board. Dentary highlighted in red to show the substantial distance between the hemi-dentary tips. (B) The ventral side of the snake jaw showing the substantial distance between the ventral head scales, mimicking the lower jaw of the corn snake during live active feeding, as seen in Figure 1B. (C) Plastic pipette tips replacing the metal clips to keep the lower jaw ‘stretched’ during microCT scanning.
